# Establishing Linkages Between Distributed Survey Responses and Consumer Wearable Device Datasets: A Pilot Protocol

**DOI:** 10.2196/resprot.6513

**Published:** 2017-04-27

**Authors:** Julia E Brinton, Mike D Keating, Alexa M Ortiz, Kelly R Evenson, Robert D Furberg

**Affiliations:** ^1^ RTI International Durham, NC United States; ^2^ Gillings School of Global Public Health Department of Epidemiology University of North Carolina Chapel Hill, NC United States

**Keywords:** Fitbit, Mturk, mHealth, clinical research protocol, consumer wearable, physical activity tracker

## Abstract

**Background:**

As technology increasingly becomes an integral part of everyday life, many individuals are choosing to use wearable technology such as activity trackers to monitor their daily physical activity and other health-related goals. Researchers would benefit from learning more about the health of these individuals remotely, without meeting face-to-face with participants and avoiding the high cost of providing consumer wearables to participants for the study duration.

**Objective:**

The present study seeks to develop the methods to collect data remotely and establish a linkage between self-reported survey responses and consumer wearable device biometric data, ultimately producing a de-identified and linked dataset. Establishing an effective protocol will allow for future studies of large-scale deployment and participant management.

**Methods:**

A total of 30 participants who use a Fitbit will be recruited on Mechanical Turk Prime and asked to complete a short online self-administered questionnaire. They will also be asked to connect their personal Fitbit activity tracker to an online third-party software system, called Fitabase, which will allow access to 1 month’s retrospective data and 1 month’s prospective data, both from the date of consent.

**Results:**

The protocol will be used to create and refine methods to establish linkages between remotely sourced and de-identified survey responses on health status and consumer wearable device data.

**Conclusions:**

The refinement of the protocol will inform collection and linkage of similar datasets at scale, enabling the integration of consumer wearable device data collection in cross-sectional and prospective cohort studies.

## Introduction

The increasing variety, functionality, and storage capacity of consumer wearable devices has created an opportunity for using the data collected on these devices for research purposes. Consumer wearables are most commonly worn on the wrist but may also be worn on clothing, at the waist, or as part of eyewear or earwear. In this paper, we focus on activity trackers, a type of consumer wearable which can collect a variety of data including steps, distance, physical activity, calories burned, quality and duration of sleep, heart rate, and location [1]. Researchers would benefit from being able to remotely gather and link these data without the need for face-to-face interaction, encouraging the use of the respondent’s own devices in the data collection process.

A remote data collection protocol would reduce the cost of providing devices to participants, reduce the time spent meeting and training respondents on their use, and increase the speed at which data could be collected [2]. Furthermore, with the ability to efficiently collect health data remotely, hospitals, worksites, and other health care providers could monitor participants in real time, reducing the need for frequent check-ups and the financial strain that is associated with those appointments [3]. The utility and success of remote-access interventions was demonstrated to be effective in collecting biometric (eg, continuous heart rate, sleep, and other health indicators) [4] data, yet research using consumer wearable devices is limited and presents challenges.

While research investigating the viability and functionality of consumer wearables has increased in recent years, thanks in part to calls for research from the National Institutes of Health and United Nations International Children's Fund, challenges still exist in the utility of these devices as the most efficient and useful way to learn about the health of an individual. Frequently, remote data collection may be hindered by burdensome notification systems, forcing individuals to use study-provided devices they are not comfortable with and requiring frequent face-to-face contact with participants in order to download data from their devices. For example, many present studies employ experience sampling methodology, which requires that researchers notify participants throughout the day requesting that they provide their remote data, a burdensome process for both respondents and researchers [5,6,7]. They often frequently require that participants travel to the researcher’s location in order to allow for the data to be downloaded. Furthermore, in regard to biometric tracking data, researchers are often required to call upon the services of a third-party software company to extract the data because there currently lacks a system that allows remote access to consumer devices for data extraction purposes [8]. Finally, many health-related research studies intend to collect data from a variety of mediums including physiologic data such as heart rate and self-reported questionnaire data such as how participants view their health. The result is a cumbersome data collection process that does not allow for a smooth data acquisition and linkage process of data from varying modes of collection.

This study seeks to explore a protocol whereby physical activity and health-related data are collected remotely through the use of personally owned activity trackers without the need for a face-to-face meeting with the respondents and without the use of study-provided devices. The primary aim of the study will be determining the feasibility of the proposed data collection protocol using an activity tracker and specifically if we are able to pair consumer wearable physiological data (ie, information from a Fitbit activity tracker) together with self-reported questionnaire data in order to have a better understanding of the health of respondents.

## Methods

### Ethics

Prior to initiation, this study will be reviewed and approved by the RTI International Institutional Review Board.

### Participant Eligibility Criteria

This study will make use of a panel of Mturkers via Amazon’s Mechanical Turk (Mturk) platform. Mturkers are a workforce of individuals willing to participate in online research studies in exchange for money deposited into their Amazon.com personal account. With the help of Mturk Prime, this study will use Mturkers as a basis for the sample. Mturk Prime operates as a team of online support staff who assist researchers in managing, communicating with, and collecting data from Mturkers (www.turkprime.com). Research using Mturkers has increased dramatically in the past few years due to the low cost and vast acquisition of data [9]. Additionally, studies show that Mturkers are more demographically diverse than standard convenience samples and samples from other online forms of data collection such as Twitter [10] and may be generalizable to the greater population [11,12]. In order for an Mturker to become a part of this study’s panel, the person will be required to either keep track of their own weight, diet, or exercise routine or keep track of their own blood pressure, blood sugar, sleep patterns, headaches, or some other health-related indicator. An eligible Mturker must also be at least 18 years of age, regularly wear a Fitbit, and be willing to give the research team access to their Fitbit data for the previous month and the upcoming month from the date of sign-up.

### Study Design and Procedure

The sample will consist of 30 Mturk participants. Participants will read a short task description and compensation information on Mturk’s research studies advertisement page. Interested participants will be asked to click a link directing them to a series of eligibility questions. If they qualify for the study based on their answers, participants will complete an electronic informed consent and become part of the Mturk Prime panel. All panelists will receive a unique numeric participant ID. Once the panel is confirmed, all 30 panelists will complete a health questionnaire that assesses demographics, general health, physical activity, health tracking processes, and consumer technology (see Multimedia Appendix 1 for full questionnaire specifications including skip logic and response codes). Participants will be asked to enter their unique participant ID into a text box at the start of the questionnaire. At the end of the questionnaire, participants will be queried for their willingness to allow researchers to download their Fitbit data. All varieties and models of Fitbit will be allowed in this study (a range of devices is summarized in the research of Evenson et al [13]).

**Figure 1:**
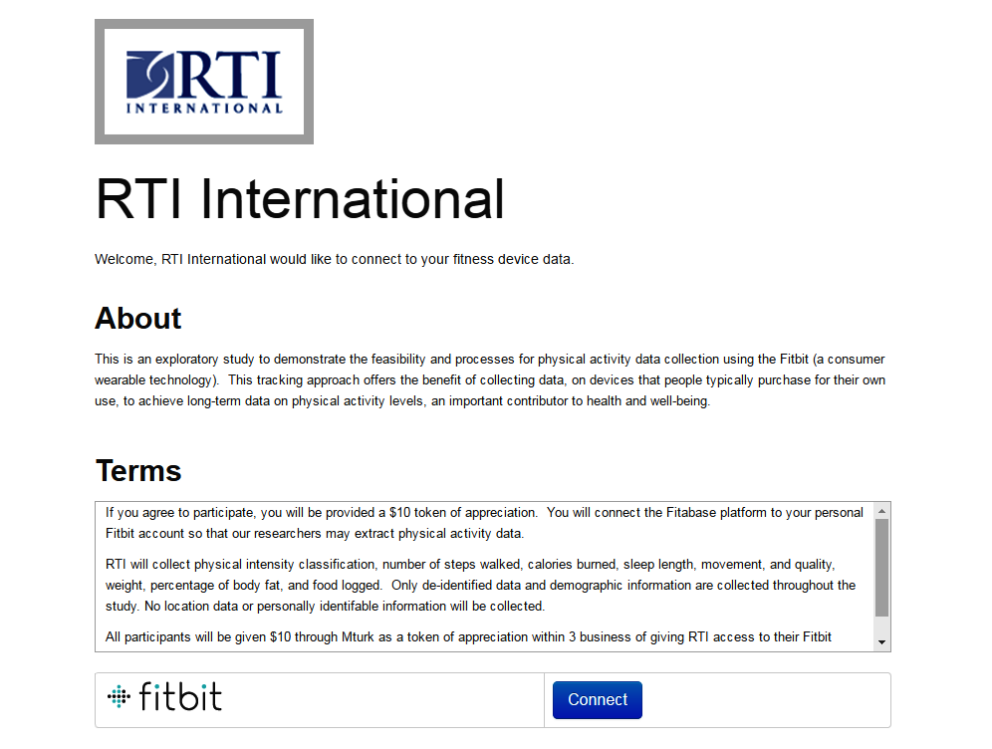
Example screen showing the Fitabase linkage.

Upon consent to Fitbit data access, participants will be routed to a third-party data service provider called Fitabase LLC (San Diego, California). Using the Fitbit application programming interface, third-party services such as Fitabase can access and aggregate self-tracker data. Fitabase provides researchers with a connection to the Fitbit infrastructure to support data collection. The research team will generate unique Fitabase links for each participant. When respondents reach the end of the self-administered survey, they will click on the link to Fitabase that corresponds to their participant ID (Figure 1). Upon completion of the Fitabase sign-up, participants will be given $10 via Mturk Prime’s customer service team. Participants who complete the questionnaire but do not sign up with Fitabase will not receive the $10 incentive. Participants can refuse to participate or cancel registration at any time. We will contract with Fitabase to provide the research team with 30 days of retrospective data and 30 days of prospective data, both from the date of sign-up. After the 30 days of prospective data are complete, Fitabase will terminate the connection to the individuals’ Fitbit device.

### Study Proposed Variables

Fitabase will be used to extract daily and intraday data from the linked Fitbit accounts. These variables include daily-level data on total steps, distance, calories burned, total sleep time, and daily active versus sedentary time. We will also obtain hourly-level data on calories burned, active versus sedentary time, heart rate, sleep, and step counts. The most granular output, intraday data, will include minute-level step counts. These data will be downloaded as both raw and aggregated files by day, per person.

### Study Outcomes and Data Analysis

#### Overview

This study’s main goal is to test the feasibility of extracting personal Fitbit data from remote survey respondents with whom the research team will never have direct face-to-face contact and then linking the biometric data to self-reported questionnaire health data. Ease of contact, maintenance, troubleshooting, and collecting participant Fitbit data throughout the study will be a vital determinant of success. More specifically, the success of the protocol will be measured by the ability to collect data from participants without face-to-face contact and high costs but with fast data acquisition and the ability to easily contact participants if there is any trouble with data collection or incentive payment.

Future analyses are planned for the physiological and self-administered data to be collected throughout the study. The following data management and data analysis plans briefly outline the proposed future acquisition, management, and analysis of these data.

#### Data Management

Study IDs will be assigned to each participant. The consumer device account identifiers will then be mapped to the assigned IDs. Once the survey is complete, the responses will be exported to comma-separated value (CSV) files. Separately, the consumer wearable datasets will be processed and sent to the researchers from Fitabase. Both the survey responses and the consumer wearable dataset will be merged by ID as a de-identified, compressed CSV file and formatted for analysis.

#### Data Analysis

Descriptive statistics will be generated for each variable. We do not expect missing data to be an issue but will explore as appropriate. The variability in Fitbit device type will be described, as well as the type, quality, and fidelity of the data collected. We will explore the Fitbit results with self-reported characteristics such as how steps per day vary by gender and age.

## Results

Data collection was conducted between April 11, 2016, and May 12, 2016. Data analysis will take place in 2017.

## Discussion

### Summary

This study provides a unique and innovative protocol for remote data collection using a common physical activity tracker. The study will be cost effective and easily manageable in that researchers do not need to meet with participants face-to-face at any point in the study and participants are able to use their own personal device to participate in the study.

With the acquisition of these data, we will be able to learn detailed information about the health of these individuals without meeting the participant face-to-face for an interview or in-person physiological assessments. Furthermore, we will learn more about the ease at which participants navigated the questionnaire-to-Fitabase linkage system by determining what proportion of participants were able to complete the online questionnaire but were unable to connect their personal Fitbit to the Fitabase platform. Finally, these data will indicate the frequency at which users sync their Fitbit, allowing us to learn more about the normal use and wearing habits of Fitbit users.

### Limitations

This feasibility study has several limitations. First, this study is targeted to a specific population, and Mturkers may not be generalizable to other populations. However, Mturkers are familiar with the online environment and therefore may be more adept at performing tasks with technology, thus making feasibility of the protocol administration more likely to be successful.

Second, this study will require respondents to have access to a Fitbit device in order to participate. Individuals who can afford and use Fitbit devices and other consumer wearables are more likely to be younger (between the ages of 18 and 34 years) and affluent [14], thus impacting generalizability. Third, the initial process of gathering a panel of participants will require respondents to complete a screener and then at a later date, complete a questionnaire in order to allow researchers to choose a varied participant pool who all use Fitbit devices. A more streamlined process would be preferred in future studies whereby participants would be able to fill out the screener and immediately begin the questionnaire if they are eligible, without the need to create a panel of participants. Unfortunately, this study will not be able to employ this methodology due to panel restrictions.

### Conclusion

This study will demonstrate that activity tracker data (ie, Fitbit data) can be remotely gathered from participants without face-to-face contact and with the use of respondent’s personal consumer wearable devices. Future research could investigate the feasibility of remote data collection without the need of a 2-step data management process as well as assess the clinical validity of consumer wearable devices, like Fitbit, to ensure that the data are accurate. If effective, this methodology could be used as a guide for researchers to implement when setting up a remote data collection system and could be applied to other consumer wearable devices as well.

## Appendix

Multimedia Appendix 1 Survey specifications for questionnaire to assess demographics, general health, physical activity, health tracking processes, and consumer technology adoption.

## Abbreviations

CSV: comma-separated values
Mturk: Mechanical Turk
