# Supplementary material for: Establishing Linkages Between Distributed Survey Responses and Consumer Wearable Device Datasets: A Pilot Protocol
Source: JMIR Res Protoc. 2017 Apr 27;6(4):e66. doi: 10.2196/resprot.6513 (PMC5427248; doi:10.2196/resprot.6513)
Supplement: Multimedia Appendix 1 [file resprot_v6i4e66_app1.pdf]

MULTIMEDIA APPENDIX 1: Survey specifications for questionnaire to assess demographics, general health, physical activity, health tracking processes, and consumer technology adoption

ConsentForm

F2b. Did you consent to participate?
1 – yes
2 – no

If no, user exits survey

Section 1: Demographics
D1. What is your gender?
1 - Male
2 – Female
3 - Other

D2. What is your race/ethnicity?
1 – White
2 – Black or African American
3 – American Indian or Alaska Native
4 – Asian
5 –Native Hawaiian or Pacific Islander
6 – Other, specify

D2a. Are you of Hispanic, Latino, or Spanish origin?
1 – Yes
2 – No

D3. What is your age?
1 - 18-29
2 - 30-49
3 - 50-64
4 - 65+

D4. What is your highest level of education?
1 - Less than high school
2 - High school graduate
3 - Some college
4 - College graduate
5 - Graduate degree

D5. What is your annual household income?
1 - Less than $30,000
2 - $30,000-$49,999
3 - $50,000 - $74,999
4 - $75,000+

Section 2: Health
H1. Would you say that in general your health is:
1 - Excellent
2 - Very good
3 - Good
4 - Fair
5 - Poor
6 - Don't know / Not sure

H1a. During the past month, other than your regular job, did you participate in any physical activities or exercises such as running, calisthenics, golf, gardening, or walking for exercise?
(91)
1 Yes
2 No
7 Don't know / Not sure

H2. Are you now living with any of the following health problems or conditions?

	Yes	No
a. Diabetes or sugar diabetes		
b. High blood pressure		
c. Asthma, bronchitis, emphysema, or other lung conditions		
d. Heart disease, heart failure or heart attack		
e. Cancer		
f. Any other chronic health problem or condition not listed		

H3. In the last 12 months, have you personally...

	Yes	No
a. Faced a serious medical emergency or crisis		
b. Gone to the emergency room or been hospitalized unexpectedly		
c. Experienced any significant change in your physical health, such as gaining or losing a lot of weight, becoming pregnant, or quitting smoking		
d. Experienced a major diagnosis (e.g., cancer)		

Section 3: Tracking

T1. What type(s) of phone(s) do you currently own? Select all that apply.
1 – Landline phone
2 – Cellular phone (includes smartphones)
3 – I do not own a phone.

T2. Now thinking about your health overall... Do you currently keep track of your own weight, diet, or exercise routine, or is this not something you currently do?
1- Yes, keep track
2 - No, not something I currently track
99- Don't know

T3. How about any other health indicators or symptoms? Do you happen to track your own blood pressure, blood sugar, sleep patterns, headaches, or any other indicator?
1 - Yes
2 - No
99- Don't know

T4. [If T1=2] On your cell phone, do you happen to have any software applications or "apps" that help you track or manage your health, or not?
1 - Yes
2 - No
99 - Don't know

T5. [If T4=1] What health issue(s) do your apps help you manage? Please select all that apply.
1 – Physical activity or exercise routines, (includes specific types of exercise like running, ab workouts, yoga, etc.)
2 - Diet, food, calorie counter
3 - Weight
4 - Period or menstrual cycle
5 - Blood pressure
6 - Heart rate monitoring
7 - Pregnancy
8 - Blood sugar
9 - Medication adherence
10 - * Mood *
11 - Sleep
12 - Other (SPECIFY)
99 - Don't know

Section 4: Tracking Health Indicators [If T3=1 or if T2=1]
T6. [If T3=1 or T2=1] Thinking about the health indicator you pay the MOST attention to, how do you keep track of changes? Do you use…
Please select all that apply.
1 - Paper, like a notebook or journal
2 - A computer program, like a spreadsheet
3 - A website or other online tool
4 - An app or other tool on your phone or mobile device
5 - A medical device, like a glucose meter
6 - Or do you keep track just in your head?
7 - Other (SPECIFY)
99 - Don't know

T7. [If T3=1 or T2=1] How often do you update your records or notes about this health indicator? Do you do this on a regular basis, or only when something comes up or changes?
1- On a regular basis
2- Only when something comes up or changes
99 - Don't know

T8. [If T3=1 or T2=1] Do you update this information…
1 - Several times a day
2 - About once a day
3 - 3-5 days a week
4 - 1-2 days a week
5 - Once or twice a month
6 - Less than once a month

99 - Don’t know 1

T9. [If T3=1 or T2=1] Do you share these health tracking records or notes with anyone, either online or offline?
1 - Yes
2 - No
99 - Don't know

T10. [If T3=1 or T2=1 and T9=1] Who do you share this information with? Please select all that apply.
1 - Health or medical professional (includes doctor, nurse, therapist, physician's assistant)
2 - A spouse/partner
3 - Parent
4 - Child
5 - Brother/sister/sibling
6 - Other Family member/Family relationship
7 - Friend
8 - Member of Group: Church, community association, volunteer group
9 - Personal trainer or health coach
10 - Other (SPECIFY)
99 - Don't know

T11. [If T3=1 or T2=1] In which of the following ways, if any, has tracking this health indicator affected your own health care routine

	Yes	No	Not applicable
a. Affected a decision about how to treat an illness or condition			
b. Changed your overall approach to maintaining your health			
c. Led you to ask a doctor new questions, or to get a second opinion from another doctor?			

Section 4: Fitbit

F1. Which of the devices do you currently use to track your fitness, steps, or physical activity? Please select all that apply.
1 - Fitbit
2 - Jawbone
3 - Nike FuelBand
4 - Android Smartwatch
5 - Apple Watch
6 - Other (specify)
7 – None of the above
99 - Don't know

F2.
Would you be willing to give the research team temporary access to your Fitbit activity data? These data are limited to physical intensity classification; number of steps walked; calories burned; sleep length, movement, and quality; weight; percentage body fat; and any food logged. You will receive $10 in compensation.
1 - Yes
2 – No

F2a [If F2 = 2Thank you. Why did you choose not to give the research team access to your Fitbit activity data?
[Open ended]
2 - Prefer not to answer

FitabaseLink [If F2b=1] Thank you. Please click on this link to connect the Fitabase application to your Fitbit account.
